# The combination of symphysis-fundal height and abdominal circumference as a novel predictor of macrosomia in GDM and normal pregnancy

**DOI:** 10.1186/s12884-020-03157-7

**Published:** 2020-08-12

**Authors:** Zhi Guo Chen, Ya Ting Xu, Lu Lu Ji, Xiao Li Zhang, Xiao Xing Chen, Rui Liu, Chao Wu, Yan Ling Wang, Han Yang Hu, Lin Wang

**Affiliations:** 1 Department of Histology and Embryology, Wuhan University, School of Basic Medical sciences, 185 East Lake Road, Wuhan, Hubei 430071 People’s Republic of China; 2 Department of Human Anatomy, Basic Medical sciences of Xinxiang Medical University, Xinxiang, 453003 China; 3 Department of Ultrasound Imaging, Zhongnan Hospital of Wuhan University, Wuhan, 430071 China; 4 Hubei Provincial Key Laboratory of Developmentally Originated Disease, Wuhan, 430071 China

**Keywords:** Abdominal circumference (AC), Gestational diabetes mellitus (GDM), Macrosomia, Symphysis-fundal height (SFH)

## Abstract

**Background:**

Macrosomia is a major adverse pregnancy outcome of gestational diabetes mellitus (GDM). Although BMI, symphysis-fundal height (SFH) and abdominal circumference (AC) are associated with foetal weight, there are some limitations to their use, especially for the prediction of macrosomia. This study aimed to identify a novel predictive methodology to improve the prediction of high-risk macrosomia.

**Methods:**

Clinical information was collected from 3730 patients. The association between the ISFHAC (index of the SFH algorithm multiplied by the square of AC) and foetal weight was determined and validated. A new index, the ISFHAC, was evaluated by area under the curve (AUC) analysis.

**Results:**

A total of 1087 GDM and 657 normal singleton pregnancies were analysed. The ISFHAC was positively correlated with foetal weight in GDM pregnancies and normal pregnancies (NPs). The AUCs of the ISFHAC were 0.815 in the GDM group and 0.804 in the NP group, which were higher than those of BMI, SFH, AC and GA. The ISFHAC cut-off points were 41.7 and 37 in the GDM and NP groups, respectively. The sensitivity values for the prediction of macrosomia with high ISFHAC values were 75.9 and 81.3% in the GDM and NP groups, respectively, which were higher than those with BMI. Regarding the validation data, the sensitivity values for prediction with high ISFHAC values were 78.9% (559 GDM pregnancies) and 78.3% (1427 NPs).

**Conclusions:**

The ISFHAC can be regarded as a new predictor of and risk factor for macrosomia in GDM pregnancy and NP.

## Background

The increasing prevalence of overweight/obesity during pregnancy has increased the risk of adverse pregnancy outcomes by increasing the prevalence of GDM. Gestational diabetes mellitus (GDM) is defined as glucose intolerance that is first diagnosed during pregnancy [1]. Women with GDM have high levels of blood glucose and insulin-like growth factor (IGF). IGF-1 plays an important role in regulating foetal growth. In the relatively long period of pregnancy, foetuses are in a state of rapid growth and require high levels of nourishment, especially in middle and late pregnancy. Nutritional counselling and exercise intervention are suitable non-invasive therapeutic options that can be readily applied to manage weight gain and improve pregnancy outcomes in women with GDM [2–6]. The incidence of foetal macrosomia and caesarean delivery is also significantly higher for GDM pregnancies [7]. Foetal macrosomia, defined as a birth weight ≥ 4000 g, affects 12% of newborns from non-GDM pregnancies and 15–45% of newborns from GDM pregnancies [8, 9]. Thus, in the context of GDM, foetal growth assessment is an important part of antenatal care. BMI, obesity and waist circumference are the conventional risk factors for pregnancy outcomes. B-ultrasonography and symphysis-fundal height (SFH) and abdominal circumference (AC) measurements are monitoring approaches that are used routinely in departments of obstetrics and gynaecology. Several studies have revealed that BMI, obesity, SFH and AC are associated with foetal weight, and these parameters are commonly used to predict foetal size and select a safe delivery method [10–12]. However, BMI, SFH and AC are not powerful enough for the diagnosis of macrosomia [13]. The aim of this study was to develop a new index to predict macrosomia. The index of symphysis-fundal height and abdominal circumference (ISFHAC) combines SFH and AC, which are used to evaluate foetal birth weight, and this index has great potential for use in predicting macrosomia in normal pregnancies (NPs) and GDM pregnancies.

## Methods

### Study design

This prospective study was conducted from 2013 to 2016 at the Department of Obstetrics and Gynecology in Zhongnan Hospital of Wuhan University. All participants provided informed written consent prior to taking part. The consent process, together with all other aspects of the study, was approved by the Research Ethics Committee of Wuhan University.

**Fig. 1:**
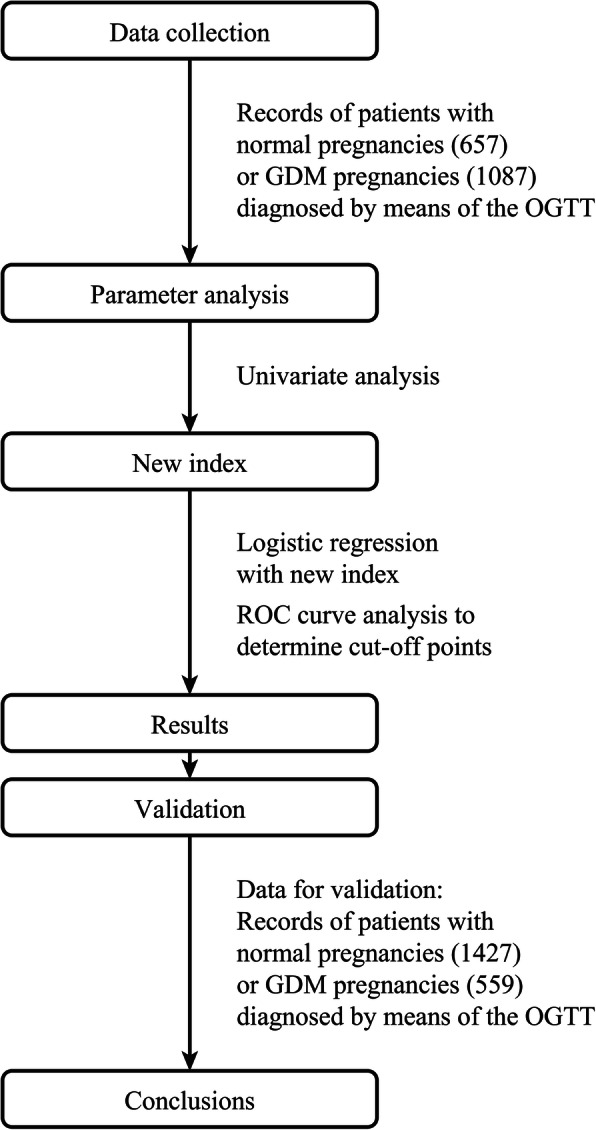
Flow chart of selection of participants included in the study

The methods and procedures for this prospective study were designed. The objectives were addressed with two sets of patients. One set, composed of 1744 patients, was used to analyse the relationship between several clinical parameters (abdominal circumference, symphysis-fundal height, BMI and gestational age) and macrosomia in the NP group and GDM group. According to the above results, a new index was obtained, and ROC curve analysis was carried out with the ISFHAC and macrosomia to determine the cut-off points for NP and GDM pregnancy. Subsequently, the other set, composed of 1986 patients, was used to validate the results (Fig. 1).

### Participants

Pregnant women who were ≥ 16 years old and had a singleton pregnancy were recruited. All target audiences were identified according to the inclusion criteria in the WHO 2013 guidelines [14]. All patients received standard prenatal check-ups between 24 and 28 weeks. The study group was divided into GDM and NP groups with an OGTT. If a patient developed GDM, an intervention, such as physical activity and dietary behavioural intervention, was begun [15]. Formal systematic testing for gestational diabetes is usually performed between 24 and 28 weeks of gestation. A standard OGTT is performed after overnight fasting by giving 75 g of anhydrous glucose in 250–300 ml water. GDM is diagnosed at any time in pregnancy if one or more of the following criteria are met: fasting plasma glucose 5.1–6.9 mmol/l (92–125 mg/dl); 1 h plasma glucose ≥10.0 mmol/l (180 mg/dl) following a 75 g oral glucose load; 2 h plasma glucose 8.5–11.0 mmol/l (153–199 mg/dl) following a 75 g oral glucose load.

In addition, the exclusion criteria were as follows: (1) age < 16 years old; (2) multiple pregnancy; (3) hypertension; (4) assisted fertilization; and (5) pregestational diabetes mellitus.

### Procedures

Data on characteristics including past medical history of hypertension, pregestational diabetes mellitus and preeclampsia, family history of DM and information before delivery, such as gestational age (GA), age, parity, prepartum height and weight, and mode of delivery, was collected. SFH, AC, and foetal birth weight were collected for each study group. The validation set was mainly missing prepartum BMI data, but the other clinical data were sufficient.

BMI data were collected on the day before delivery. BMI calculations are routinely performed before childbirth in patients who appear underweight, overweight or obese. Categorical definitions of normal weight (BMI < 25), overweight (BMI 25 to < 30) and obesity (BMI ≥ 30) follow the recommendations of the World Health Organization.

Gestational age was based on the last reliable normal menstrual period and confirmed by ultrasound foetal biometry before 20 weeks or by ultrasound dating performed before 20 weeks. GA was a major clinical parameter identified by doctors from the information of the pregnant women.

Infant weight was measured by an obstetrician or midwife after delivery. Weight assessment was measured with a baby scale, and nude weight data were obtained. These data contained two decimal places. To improve the accuracy of this parameter, repeated measurements were performed by averaging the testing values. Foetal macrosomia, defined as a birth weight ≥ 4000 g.

SFH was measured from the superior border of the symphysis to the highest uterine fundus. The tape was positioned with one hand over the upper border of the pubis symphysis bone, and the tape was placed in a straight line over the uterus until loss of resistance was felt when reaching the fundus. The tape was turned so that the numbers were visible to record the value to the nearest complete one centimetre. The SFH was measured by a doctor at the prenatal check-up. The last SFH measurement was antepartum.

AC was defined as the length of the abdominal circumference through the umbilicus. Data were determined by measuring the abdominal circumference with a measuring tape.

We assumed that a pregnant woman’s abdomen was cylindrical; if pregnant women’s abdomens are an ideal cylinder, we know that the volume is equal to the basal area times the height. According to the density calculation formula, we can obtain the weight. This parameter served as a new index that predicted foetal weight. The new index was calculated with the formula below: ISFHAC $=\frac{\rho }{4\pi }$ ×SFH (m) × AC (m)^2^, where ρ is the density of the human body. $\frac{\rho }{4\pi }$ is taken as a constant that is approximately equal to 100. Thus, the ISFHAC was calculated to predict macrosomia.

First, logistic regression was used to analyse the relationship between macrosomia and the data parameters. Then, ROC curve analysis was carried out with the ISFHAC and macrosomia. We determined the cut-off points for an NP and GDM pregnancy. According to the following cut-off point, the samples were divided into high and low index groups: less than the cut-off points was the low index group and greater than the cut-off point was the high index group in the GDM and NP groups. To verify the predictive value of the ISFHAC for macrosomia, the ISFHAC was applied in the analysis set (1744) and the validation set (1986).

### Statistical analysis

Statistical analysis was performed using IBM SPSS Statistics version 20.0. For measurement data, an independent-sample t test was used. Afterward, ROC curve analysis was performed with the ISFHAC and macrosomia. We determined the capacity of the ISFHAC for predicting macrosomia by univariate logistic regression analysis. To compare the effect of the predictive value of BMI for macrosomia, we examined the effect of its prediction of macrosomia in the NP and GDM groups in the analysis cohort. Sensitivity, specificity and accuracy were determined in the analysis.

## Validation of the prediction model

To further evaluate the predictive effect of the ISFHAC for macrosomia, data from another 1986 additional patients were used for the clinical trial evaluation. Sensitivity, specificity and accuracy were estimated.

## Results

### Study characteristics

According to the data we collected, the ages of the participants ranged from 16 to 54, and women with GDM were older than women in the control group. The mean prenatal BMI in the GDM group was higher than that in the control group. Obesity in the GDM group was 3.27-fold that in the control group, while the number of patients with normal weight in the control group was 2.53-fold that in the GDM group. There were statistically significant differences in BMI categories between the control and GDM groups. The ratio of multiparas with GDM was 2.17-fold that of multiparas in the NP group, and the family history of DM was higher in the GDM group than in the control group. The percentage of caesarean sections was 75.6% in the GDM group, which was higher than that in the control group (69.9%) (Table 1).

**Table 1:** Characteristics of NP and GDM pregnancy groups

	NP (*N* = 657)	GDM (*N* = 1087)	*P*-value
Mean (SD) age at delivery (y)	28.86 (3.96)	29.62 (4.76)	*P* = 0.001 ^a^
Mean (SD) gestational weeks (w)	38.81 (1.80)	38.91 (1.22)	*P* = 0.175^a^
Women with BMI			
Mean (SD) BMI (kg/m^2^)	26.36 (3.04)	29.1 (3.95)	*P* < 0.0001^a^
Pregnancy BMI categories (%)			*P* < 0.0001^b^
≥ 18.5& < 25	213 (32.4%)	139 (12.8%)	
≥ 25& < 30	358 (56%)	536 (49.3%)	
≥ 30	76 (11.6%)	412 (37.9%)	
Parity (%)			*P* < 0.0001^b^
Nulliparous	535 (81.4%)	649 (59.7%)	
Parous	122 (18.6%)	438 (40.3%)	
Family history (%)			*P* < 0.0001^b^
No DM	646 (98.3%)	954 (87.8%)	
With DM	11 (1.7%)	133 (12.2%)	
Mode of delivery (%)			*P* = 0.009^b^
Vaginal	195 (30.1%)	266 (24.4%)	
Cesarean section	453 (69.9%)	824 (75.6%)	

*GDM* gestational diabetes mellitus, *BMI* body mass index

Regarding pregnancy BMI categories, ≥18.5& < 25 means normal weight; ≥25& < 30 means overweight; ≥30 means obesity. Regarding gestational weeks, < 37 means premature birth; ≥37& < 42 means mature birth; ≥42 means postterm birth

^a^*p* values were calculated using the independent sample T-test, and

^b^*p* values were calculated using the chi-square test

### The ISFHAC is a novel potential predictor of macrosomia

**Fig. 2:**
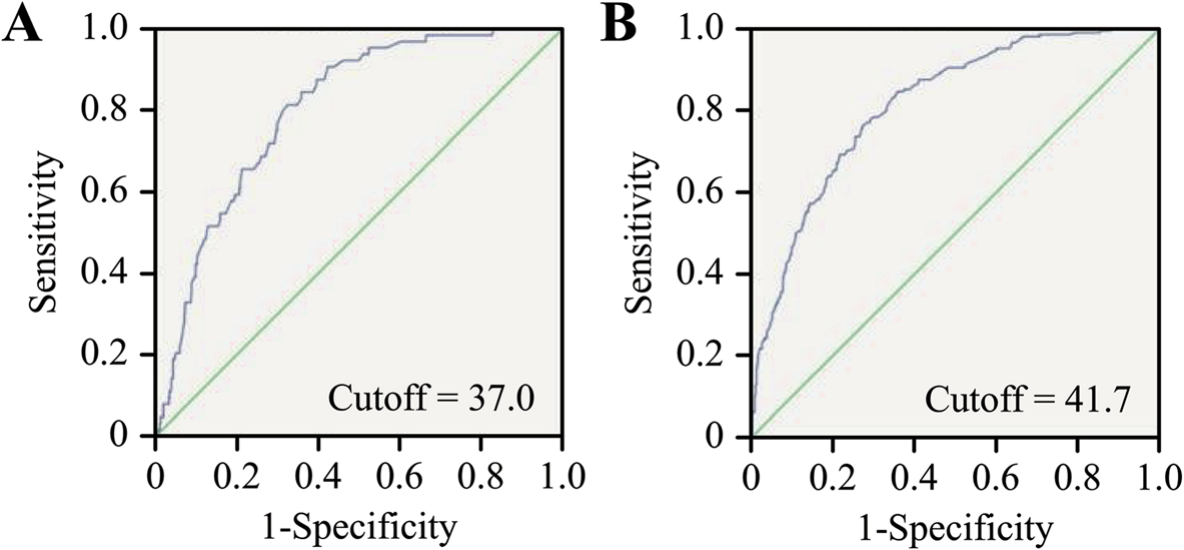
The AUC of the ISFHAC in the control and GDM groups. **a** The area under the ROC curve of the control group was 0.803, and the cut-off point was 37.0. **b** The area under the ROC curve of the GDM group was 0.815, and the cut-off point was 41.7

As shown in Supplementary Table 1, the logistic regression analysis indicated that foetal weight was associated with SFH, AC and BMI. Subsequently, to evaluate the effect of the ISFHAC in predicting the risk of macrosomia, ROC curve analysis was performed on the data for analysis and evaluation (Fig. 2). The area under the curve (AUC) of the ISFHAC was larger (area = 0.803, *p* < 0.0001) in the control group, and the AUC of the ISFHAC was larger (area = 0.815, *p* < 0.0001) in the GDM group, both of which were higher than those of BMI, SFH, AC and GA. The prediction ability of the ISFHAC is higher than that of other parameters. Therefore, we could determine the cut-off points for each group (Table 2). The ISFHAC value was higher in the GDM group than in the control group.

**Table 2:** ROC curve analysis of the utility of clinical parameters for predicting macrosomia

	GDM		NP	
	Area	*P*-value^a^	Area	*P*-value^a^
ISFHAC	0.815	< 0.001	0.803	< 0.001
SFH	0.804	< 0.001	0.767	< 0.001
AC	0.753	< 0.001	0.744	< 0.001
BMI	0.707	< 0.001	0.651	< 0.001
GA	0.540	0.07	0.640	< 0.001

^a^ *p* values were calculated by ROC curve analysis

The cut-off points for the ISFHAC in the NP and GDM groups were 37 and 41.7, respectively. The ISFHAC values were divided into three categories according to BMI. Of note, in the GDM and NP groups, 41.7 and 37, respectively, were the lower bounds for the ISFHAC according to obesity (Supplementary Table 2).

### The ISFHAC can predict macrosomia in GDM pregnancy and NP

Moreover, there were 208 and 64 cases of macrosomia in the GDM (1087, 19.1%)) and NP (657, 9.7%) groups, respectively; the rate of macrosomia in the GDM group was 2-fold that in the NP group. Samples were divided into high- and low-ISFHAC groups according to the following cut-off points: less than 41.7 as the low index group and greater than 41.7 as the high index group in the GDM group and less than 37 as the low index group and greater than 37 as the high index group in the NP group. We further predicted macrosomia in our study. The cut-off point was 41.7 in the GDM group, with a sensitivity of 75.9%, specificity of 72.9%, and accuracy of 73.5%. The cut-off point was 37 in the NP group, with a sensitivity of 81.3%, specificity of 66.4%, and accuracy of 67.9% (Table 3). A high ISFHAC value could predict 75.9% of macrosomia cases, but with BMI, the prediction for macrosomia was only 60.1% in the GDM group. In the NP group, the high ISFHAC and BMI prediction values were 81.3 and 25%, respectively (Table 3).

**Table 3:** Macrosomia with ISFHAC and BMI analysis

	GDM (*N* = 1087)		NP (*N* = 657)	
	Macrosomia (*n* = 208)	Normal (*n* = 879)	Macrosomia (*n* = 64)	Normal (*n* = 593)
ISFHAC ≥41.7/37	158 (75.9%)	238 (27.1%)	52 (81.3%)	199 (33.6%)
BMI ≥ 30	125 (60.1%)	287 (32.7%)	16 (25%)	60 (10.1%)

### Validation results

To evaluate the predictive power of the ISFHAC, 559 (GDM pregnancy) and 1427 (NP) women were screened for validation and used for the clinical trial evaluation. A high ISFHAC value could predict macrosomia in the NP group with a sensitivity of 78.9%, specificity of 71.3%, and accuracy of 72.1%. In the GDM group, the sensitivity was 78.3%, the specificity was 82.8%, and the accuracy was 82.3% (Table 4).

**Table 4:** The validation of ISFHAC for predicting macrosomia in the GDM pregnancy and NP groups

	GDM (*N* = 559)		NP (*N* = 1427)	
	Macrosomia (*n* = 60)	Normal (*n* = 499)	Macrosomia (*n* = 147)	Normal (*n* = 1280)
ISFHAC ≥41.7/37	47 (78.3%)	86 (17.2%)	116 (78.9%)	367 (28.7%)
ISFHAC < 41.7/37	13 (21.7%)	413 (82.8%)	31 (21.1%)	913 (71.3%)

## Discussion

In recent decades, China has experienced changes in dietary intake and decreased physical activity [16]. The report released in China shows that 72.1 million female patients have prediabetes. Among women between the ages of 20 and 39 years, approximately 5.6 million have DM (3.2%), and 15 million have prediabetes (9%) [17].

Regarding the specific eating habits of Chinese people and the lack of sufficient exercise during pregnancy, obesity in the GDM group was higher than that in the NP group. A previous study revealed that normal weight accounted for most NPs [18]. In this study, however, 67.6 and 87.2% of the patients in the NP and GDM groups, respectively, were overweight and obese.

A higher BMI, AC, and fasting glucose in the first trimester of pregnancy increased the GDM risk [19]. Excessive gestational weight gain, according to the targets set by the Institute of Medicine (IOM), was associated with caesarean section, LGA and macrosomia. Modification of the IOM criteria, including more restrictive targets, did not improve perinatal outcomes [20]. Our results indicated that there was a high percentage of obesity in the GDM group, that this percentage was 1.96-fold that of the control group for predicting macrosomia, and that obesity can also lead to adverse pregnancy outcomes. In addition, other groups have reported the relationship between obesity and adverse pregnancy outcomes [21].

In a previous study, the incidence of foetal macrosomia (the main outcome) was significantly higher in the GDM group (20.0%) than in the control group (3.6%) [22]. In our research, foetal macrosomia was observed in 9.7% of women in the control group and in 19.1% of women with GDM.

Antenatal care was important for the maternal and foetal outcomes, and SFH and AC are two routine measurements in obstetrical departments. They have clinical significance for predicting infant size and as a reflection of the pregnant woman’s nutritional status. These findings support the internal validation of the SFH chart, which may be implemented in the prenatal care of patients with diabetes and pregnancy [12]. However, one reference shows that there is no evidence that SFH is useful to identify macrosomia [13]. The SFH measurement is primarily used to detect foetal intrauterine growth restriction (IUGR). Undiagnosed IUGR may lead to foetal death, as well as to increased perinatal mortality and morbidity [23].

To our knowledge, this is the first time that the notion of combining SFH and AC to calculate the ISFHAC has been put forth as an indicator of pregnancy outcome.

Regarding the AUCs of different parameters, the AUC for the ISFHAC was the largest among the NP and GDM groups. Thus, we think that the relationship between the ISFHAC and macrosomia is relevant. In this study, the cut-off points for the ISFHAC were 37 and 41.7 in the control and GDM groups, respectively. Women in the high bin of the index were prone to adverse pregnancy outcomes. Interestingly, 41.7 was the lower bound of the ISFHAC, which is consistent with obesity in GDM, and 37 was the lower bound of the ISFHAC in the control group, which is also in accordance with obesity. In the analysis group, our results indicated that the ISFHAC is superior to other parameters (e.g., BMI) for predicting macrosomia. Thus, we only analysed the new index in the validation group.

We were interested in the high index group. Here, the high ISFHAC predicted (75.9%) most of the macrosomia cases in the GDM group, and this rate was higher than that of the obesity-based grouping (60.1%).

In the NP group, the high ISFHAC predicted 81.3% of macrosomia cases, and obesity predicted 25% of macrosomia cases. The high ISFHAC prediction ability for macrosomia was better than that of the obesity-based grouping.

In another validation dataset, a high ISFHAC predicted most of the macrosomia cases in the NP and GDM groups. A high ISFHAC was a risk factor for macrosomia.

All measures used should aim to prevent an excessive SFH and AC, and the high ISFHAC group needs exercise or dietary intervention. Chinese GDM prevention and treatment programmes should target overweight and obese adults with central obesity. Pregnancy SFH and AC control is an important method for reducing the risk of an adverse perinatal outcome in a subsequent pregnancy. SFH and AC are constantly used as indicators of foetal weight, but they are useless for identifying macrosomia [13]. Combining these two parameters (SFH and AC) may also have limitations. Adipose panniculus may reflect SFH and AC, which would be positively associated with obesity-related adverse pregnancy outcomes. Thus, the new index has the potential to improve our future research.

Ultrasound is not a routine examination. In addition, ultrasound measurements are routinely performed on all pregnant women at 18–22 weeks gestation as a screening tool for foetal anomalies. A simple clinical risk score may help obstetricians predict macrosomia at the time of delivery in remote areas where antenatal care services are less than adequate [24].

There may be some limitations in this study. Although this study includes a large sample size, it contains only patients from a single tertiary hospital and thus cannot represent the total population. Future studies need to determine the effects of various factors, for example, using different hospital data and selecting patients who choose different occupations from different regions.

Consequently, this study provides evidence that the ISFHAC is more strongly associated with the risk of macrosomia than BMI. It is possible that the ISFHAC might be useful as a surrogate for developing adverse pregnancy outcomes, such as in predicting macrosomia. To further confirm our results, future studies are warranted to predict foetal weight in different GA groups. We hope to provide an ISFHAC chart using the index at different GAs to predict foetal weight.

## Conclusion

In this study, a new index ISFHAC was identified to improve the prediction of high-risk macrosomia in GDM pregnancy and NP. ISFHAC are used to evaluate foetal birth weight, which is superior to BMI, SFH, AC and GA. Therefore, the ISFHAC can be regarded as new clinical diagnostic criteria for prediction macrosomia, and its clinical practice utility is depending on further investigations.

## Supplementary information

**Additional file 1 Table 1.** Univariate Logistic Regression Analysis Between Macrosomia and the Maternal Clinical Parameters. **Table 2.** The mean and 95% CI of ISFHAC with BMI

## Data Availability

The datasets generated and analyzed during the current study are not publicly available due to the hospital policy but are available from the corresponding author on reasonable request.

## Abbreviations

ISFHAC: The index of SFH algorithm multiplied by the square of AC
GDM: Gestational diabetes mellitus
DM: Diabetes mellitus
BMI: Body mass index
OGTT: Oral glucose tolerance test
WHO: World Health Organization
AC: Abdominal circumference
SFH: Symphysis-fundal height
IUGR: Intrauterine growth restriction
GA: Gestational age
AUC: Area under the curve
