# Supplementary material for: The combination of symphysis-fundal height and abdominal circumference as a novel predictor of macrosomia in GDM and normal pregnancy
Source: BMC Pregnancy Childbirth. 2020 Aug 12;20:461. doi: 10.1186/s12884-020-03157-7 (PMC7425134; doi:10.1186/s12884-020-03157-7)
Supplement: Supplementary file 1 — Additional file 1 Table 1. Univariate Logistic Regression Analysis Between Macrosomia and the Maternal Clinical Parameters. Table 2. The mean and 95% CI of ISFHAC with BMI [file 12884_2020_3157_MOESM1_ESM.docx]

**Table 1.** Univariate Logistic Regression Analysis Between Macrosomia and the Maternal Clinical Parameters.

| Parameter | P-value | |
| --- | --- | --- |
|  | GDM | Control |
| BMI | <0.001 a | <0.001 a |
| SFH | <0.001 a | <0.001 a |
| AC | <0.001 a | <0.001 a |
| GA | 0.029 a | <0.001 a |
| Parity | 0.415 b | 0.474 b |
| Age | 0.048 a | 0.207 a |

GDM, gestational diabetes mellitus; BMI, body mass index; SFH, symphysis-fundal height; AC, abdominal circumference; GA, gestational age. a p values were calculated using the independent sample t-test and b p values were calculated using the Chi-square test.

**Table 2** The mean and 95% CI of ISFHAC with BMI.

| BMI | ISFHAC | | | | | | | |
| --- | --- | --- | --- | --- | --- | --- | --- | --- |
|  | GDM | | | | NP | | | |
|  | Mean | SD | -95% CI | +95% CI | Mean | SD | -95% CI | +95% CI |
| ≥18.5 & <25 | 31.53 | 5.19 | 30.66 | 32.40 | 32.35 | 5.65 | 31.58 | 33.11 |
| ≥25 & <30 | 37.38 | 5.22 | 36.94 | 37.83 | 35.91 | 5.99 | 35.30 | 36.53 |
| ≥30 | 46.66 | 7.92 | 45.89 | 47.43 | 40.93 | 7.07 | 39.31 | 42.54 |

For BMI, ≥18.5 & <25 means normal weight; ≥25 & <30 means overweight; ≥30 means obesity. -95% CI means the low bound of the 95% confidence interval; +95% CI means the high bound of the 95% confidence interval.
